# Emerging Concepts of Adaptive Immunity in Leprosy

**DOI:** 10.3389/fimmu.2018.00604

**Published:** 2018-04-09

**Authors:** Soumi Sadhu, Dipendra Kumar Mitra

**Affiliations:** ^1^Department of Transplant Immunology, All India Institute of Medical Sciences (AIIMS), New Delhi, India; ^2^Department of Immunogenetics, All India Institute of Medical Sciences (AIIMS), New Delhi, India

**Keywords:** polarized immunity, natural killer T cells, regulatory T cells, Th 17, programmed death-1-programmed death ligand-1

## Abstract

Leprosy is a chronic intracellular infection caused by the acid-fast bacillus, *Mycobacterium leprae*. The disease chiefly affects the skin, peripheral nerves, mucosa of the upper respiratory tract, and the eyes. The damage to peripheral nerves results in sensory and motor impairment with characteristic deformities and disability. Presently, the disease remains concentrated in resource-poor countries in tropical and warm temperate regions with the largest number of cases reported from India. Even though innate immunity influences the clinical manifestation of the disease, it is the components of adaptive immune system which seem to tightly correlate with the characteristic spectrum of leprosy. *M. leprae*-specific T cell anergy with bacillary dissemination is the defining feature of lepromatous leprosy (LL) patients in contrast to tuberculoid leprosy (TT) patients, which is characterized by strong Th1-type cell response with localized lesions. Generation of Th1/Th2-like effector cells, however, cannot wholly explain the polarized state of immunity in leprosy. A comprehensive understanding of the role of various regulatory T cells, such as Treg and natural killer T cells, in deciding the polarized state of T cell immunity is crucial. Interaction of these T cell subsets with effector T cells like Th1 (IFN-γ dominant), Th2 (interluekin-4 dominant), and Th17 (IL-17+) cells through various regulatory cytokines and molecules (programmed death-1/programmed death ligand-1) may constitute key events in dictating the state of immune polarization, thus controlling the clinical manifestation. Studying these important components of the adaptive immune system in leprosy patients is essential for better understanding of immune function, correlate(s) the immunity and mechanism(s) of its containment.

## Introduction

**Figure 1:**
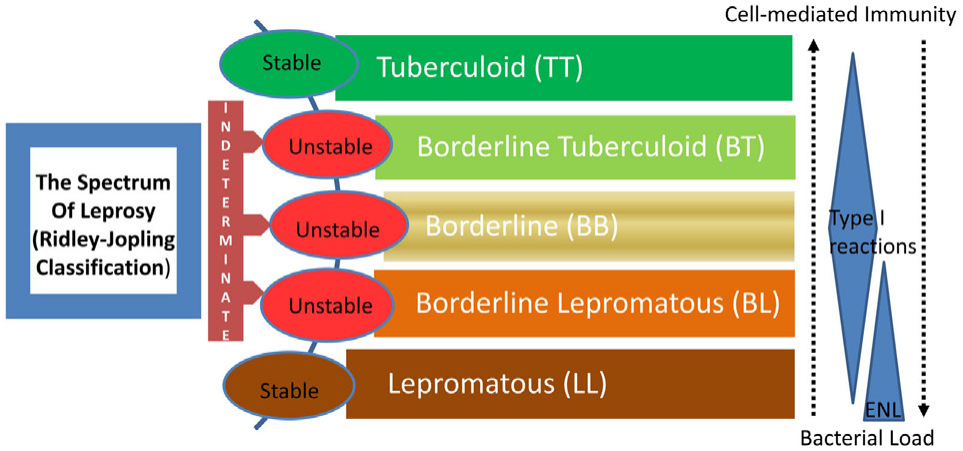
The spectrum of leprosy: Ridley–Jopling classification and the relationship with host immunity. ENL, erythema nodosum leprosum or type 2 reaction.

Leprosy is regarded as a stigmatized disease even today. Even though prevalence has fallen substantially in the past few decades, its transmission continues and the disease remains a major public health problem, especially in many third world countries. The chronic infectious disease is caused by the acid-fast, rod-shaped *Bacillus, Mycobacterium leprae*. It results in extensive damage to the skin, eyes, mucosa of the upper respiratory tract, and peripheral nerves, in some cases leading to sensory and motor impairment with characteristic deformities and disability (1). Worldwide, two to three million people are estimated to be permanently disabled because of leprosy (2). India has the largest number of cases, with Brazil second, and Burma third (2). Although the reported number of registered cases worldwide has declined in the past two decades, the number of new cases registered each year has remained almost same (3). For the immunologists, however, leprosy still garners a lot of attention mainly because *M. leprae* infection which evokes distinct polarized T cell responses in humans, which correlates with the clinical manifestations. The two polar forms of leprosy, known as tuberculoid type (TT) and lepromatous leprosy (LL), have clinical, microbiological, and immunological linkage [(4, 5), Figure 1]. TT is characterized by fewer skin lesions, low numbers of bacteria in lesions, and histologically well-formed granulomas containing abundant CD4+ T cells. On the other hand, LL is characterized by numerous infiltrative skin lesions, large numbers of bacteria in lesions, and poorly formed granulomas with fewer lymphocytes (6). However, most leprosy patients display a pathogenesis somewhere in between and are classified as either borderline tuberculoid (BT) or borderline lepromatous (BL) (4). Leprosy reactions known as type 1 reactions (T1R) (Figure 1) are common in these immunologically unstable borderline groups and involve an upregulation of the host response to *M. leprae* antigens (5). In patients with the disseminated LL, a reaction known as erythema nodosum leprosum (ENL) or type 2 reactions (T2R) is frequent, being observed in almost half of these patients receiving antimicrobial therapy (1).

## Polarized Immunity in Leprosy: Possible Causes

Several factors may be involved in regulating the polarization of newly activated naïve T cells into mature Th1 or Th2-like effector cells (7): *viz*., the local cytokine milieu; the presence of immunologically active hormones; the dose and route of antigen administration; the type of antigen-presenting cell stimulating T cells; and the “strength of signal” of the T-cell receptor for the MHC-antigen complex. The most important among these is the cytokine milieu surrounding the newly activated T cell. In the context of leprosy, reciprocal changes in cytokine expression in TT vs. LL along with complex cytokine regulatory networks have been evidenced at the site of infection (8). However, the important question is: which of these factors serve as the initial determinant of the polar immune responses to *M. leprae*? Given the extremely high relatedness of leprosy bacilli genomes worldwide (9), bacterial diversity is unlikely. This leaves differential host responses as the most likely mechanism.

Polarized T cell response (Th1/Th2 biased) to *M. leprae* is believed to be a critical element in the pathogenesis of leprosy and its varied clinical manifestations (8). The generation of Th1 effector cells chiefly producing the cytokine interferon-gamma (IFN-γ) vs. Th2 effector cells producing interluekin-4 (IL-4) have been held primarily responsible for the polarized state of immunity. During reversal reaction, lesional sites have demonstrated presence of CD4+ *M. leprae*-responsive T cells with a polarized type 1-like phenotype (10). However, the immune response manifested at the pathologic site(s) of leprosy is an extremely complex process, particularly in the light of recently evidenced remarkable heterogeneity of T cell subsets (11). Proportional enrichment of selective T cell subsets, particularly at the pathologic sites, determines the bulk T cells response (12). The major focus of the review is on the functionality of various relatively infrequent, yet significant lymphocyte subsets, which subsequently regulate the host’s cellular immune response and consequently disease pathogenesis. Numerous smaller subsets of lymphocytes have been identified in the past few decades which play critical roles in shaping the host immunity *via* their T cell response. These include natural killer T cells (NKT), regulatory T (Treg) cells, γδ T cells, and the very recently identified regulatory B cells. These cells have been demonstrated to exert regulatory influences on the generation of various effector T cells, such as Th1, Th17, and Th9-like cells (13–15).

## The NKT Cells

First described in 1987 (16, 17), NKT cells are a unique subset of mature T cells co-expressing a semi-invariant Vα24Jα18 T cell antigen receptor (TCR)α chain and surface markers characteristic of NK cells. The semi-invariant TCR on iNKT cells recognizes glycolipids bound to monomorphic CD1d molecules. The most prominent and characteristic function of NKT cells is the early rapid production of immune-regulatory cytokines, such as IL-4, IFN-γ, and TNF-α upon their activation (18).

Earlier studies on the tissue origin and developmental pathway of iNKT cells from Taniguchi’s group (19) have suggested that iNKT cells develop extra-thymically, particularly in the liver. However, others have demonstrated that the majority of iNKT cells, like conventional T cells, are generated in the thymus (20, 21). The finding that not only peptides, but also glycolipids can serve as a source of antigen recognized by these NKT cells opened up new vistas in the study of antigen processing and presentation (22). The ability of nonpolymorphic CD1 molecules to present structurally diverse glycolipids to T cells has generated interest on these fascinating lipid–protein interactions. Since, NKT cells exercise a determining influence on a variety of immune responses in mice, ranging from autoimmunity to tumors and infections (23–25), significant interest has been generated to study their roles in human diseases as well.

Invariant iNKT cells (26, 27), which have a limited diversity of their TCR chains recognize glycolipid antigens from certain bacteria that are presented by CD1d, a nonpolymorphic antigen-presenting molecule (25). CD1-restricted T cells appear to play a major role in immune responses to mycobacteria. However, results of studies in mouse models are inconsistent. For example, although CD1d-deficient mice did not differ significantly in susceptibility to *Mycobacterium tuberculosis* (28), NKT cells predominate in the granulomatous reaction to *M. tuberculosis* cell wall preparations, and such granulomas do not form in NKT cell-deficient Jα2812/2 mice (29). Furthermore, NKT cells of normal mice respond to mycobacterial infection by decreasing IL-4 and increasing IFN-γ production (30), changes that aid the host response to mycobacteria, since IFN-γ plays a critical role in pathogen clearance.

Leprosy-specific studies on NKT cells (31) have shown mycobacterium-reactive double-negative T-cell lines derived from skin lesion of a leprosy patient responded to subcellular fractions of mycobacteria in the presence of CD1-expressing antigen-presenting cells (APCs). However, lipoarabinomannan-depleted soluble cell wall fraction did not induce detectable T-cell proliferation. Recognition of purified lipoarabinomannan from *M. leprae* was restricted by CD1b, and T cells lysed lipoarabinomannan-pulsed monocytes in a CD1b-restricted manner. Lipoarabinomannan also induced these T cells to secrete large amounts of IFN-γ. Upon examination of leprosy patients, they found few CD1+ cells in LL leprosy lesions. In contrast, there was a strong upregulation of CD1+ cells in the granulomatous lesions of patients with TT leprosy or reversal reaction (32). These cells were also CD83+, a marker for dendritic cells, indicating a strong correlation between CD1 expression and cell-mediated immunity in leprosy. Interestingly, administration of granulocyte-macrophage colony-stimulating factor, a cytokine which can promote dendritic-cell activation, to LL leprosy patients induced infiltration of CD1+ cells into the lesions (33, 34). NKT cells were also found in T-cell-reactive leprosy, but when compared with the granulomas in cutaneous sarcoidosis these cells were undetectable (35). They studied the TCR Vα repertoire and found that all patients with T-cell-reactive leprosy showed a very restricted T-cell-reactive Vα repertoire with a strong bias toward the use of the Vα6 and Vα14 segments. Unpublished data from our laboratory have given clear indications that NKT cell-derived cytokines control the ensuing effector T cell responses on activation with lipid antigens and further help in dictating the overall T cell response and manifestation of the disease. All of these studies strongly suggest that NKT cells play a determining role in regulating the varied type of immune responses as evidenced in leprosy affected individuals.

## The Treg Cells

Regulatory T cells, on the other hand, are essential for maintaining peripheral tolerance, preventing autoimmune diseases, and limiting chronic inflammatory diseases (36). However, in case of chronic infections, they also limit such beneficial effect by suppressing the host immunity. During an infection, immune regulation is the result of the host’s response to the infection in a bid to maintain or restore a homeostatic environment and/or it can be actively induced by the pathogen to promote pathogen survival, like in the case of *M. leprae* (37). The presence of T cells with suppressive or anergic activity was discovered a long time back when they were known as suppressor T cells (38, 39). These same cells were shown to produce IL-10 and generated *in vivo* during infection (40). Recently, it has emerged that there are several specialized subsets of Treg cells, which contribute to the elaborate regulatory network within the infected host.

Based on their origin, generation, and mechanism of action, two main subsets of Treg cells have been identified: one is the naturally occurring CD4+CD25+ Treg cells (natural Treg cells), which mainly develop in the thymus and regulate self-reactive T cells in the periphery (41). Others are the inducible Treg cells, which develop in the periphery from conventional CD4+ T cells after exposure to signals, such as regulatory cytokines, immunosuppressive drugs, or APCs conditioned by microbial products (42). Both types of Treg cells, by virtue of their capacity to control the intensity of effector responses have been shown to have a major role in infection (12, 43). Treg cells mediate their suppressive capacity on inflammatory effector T cells, such as Th1, Th17, and Th9 cells both by contact dependent as well as contact-independent manner (36). From a functional perspective, Treg cells can be grouped into four basic “modes of action:” the various potential suppression mechanisms used by these include suppression by inhibitory cytokines, suppression by cytolysis, suppression by metabolic disruption, and suppression by modulation of dendritic cell maturation or function (44). Inhibitory cytokines, IL-10 and TGF-β, have been the focus of considerable attention as mediators of Treg cell induced suppression (45–47).

Differential trafficking of these Treg cells to the diseased sites are thought to be under the influence of tissue chemokine response elicited at the site of lepromatous lesions. This in turn is believed to determine the local immunity of BT/TT and BL/LL forms of leprosy. The tissue chemokine response at the lepromin DTH site and lesions of various forms of leprosy determines the recruitment of effector T cells at the lesional levels in leprosy patients (48). Therefore, subset composition of T cells infiltrating the pathologic/lesional site(s) of leprosy patients appears to be the key element in deciding the local immunity in leprosy, which may dictate the clinical manifestation of the disease. Some of these subsets have been demonstrated to be hierarchy in nature and known to exert significant influence on the effector T cells, and thus regulate the immune response at the pathologic site(s) of various chronic infectious diseases, including leprosy. These include the FoxP3 positive Treg cells as one of the most potent hierarchic cell type suppressing the effector T cell function with eventual regulation of immune response elicited by the host during intracellular infections, such as tuberculosis and leishmaniasis (49). Over representation of Treg cells either in peripheral compartment or more particularly at the pathologic site(s) has been shown to be of critical importance in determining the local immunity, thus dictating the outcome of the disease among patients suffering from various forms of tuberculosis (12). In leprosy as well, works have suggested that Tregs are present in increased numbers in LL patients, and they may have a pathogenic role in leprosy patients harboring uncontrolled bacillary multiplication (50). CD25+ Treg cells have also been shown to play a role in *M. leprae*-induced Th1 unresponsiveness in LL (51). FoxP3+ inducible Tregs producing the immunosuppressive cytokine TGF-β may also downregulate the T cell responses leading to antigen-specific anergy associated with LL (52).

Recent studies have revealed (53) that T2R or ENL patients have significantly lower number of circulating and *in situ* Tregs than T1R patients and controls with concomitant increase in pro-inflammatory cytokines such as TNF-α and IFN-γ produced by Th1 lymphocytes.

## The Th17 Cells

Very recently, a third subset of T helper cells, Th17 cells, has been identified based on their cytokine production profile. These cells produce IL-17A (also referred to as IL-17), IL-17F, and IL-22, cytokines involved in neutrophilia, tissue remodeling and repair, and production of antimicrobial proteins. Th17 cells differentiate in response to the STAT3-activating cytokines IL-6, IL-21, and IL-23 along with TGF-β and IL-1β (54). They are abundant at mucosal interfaces, where they contain infection with pathogenic bacteria and fungi (55). Skin-homing T helper cells that produce IL-22, but not IL-17, have also been described in humans, and they may represent a new T cell subset with distinct effector functions (56). It is believed that the differentiation of CD4+ T cells that produce IL-17 and IL-22 is influenced by the composition of the intestinal microbiota and by the presence of innate immune cells mainly the neutrophils that amplify the Th17 cell response.

For long, Th1 cells were considered to be the major effectors in multiple autoimmune diseases, while Th2 cells were involved in atopy and asthma. In recent times, however, Th17 cells have been implicated as culprits in a plethora of autoimmune and other inflammatory diseases in mice and humans. Many diseases that were previously associated with Th1 cells, e.g., experimental autoimmune encephalomyelitis (EAE, a model for multiple sclerosis), collagen-induced arthritis, and some forms of colitis, were shown to be caused by IL-23-dependent Th17 cells or other IL-17-producing lymphoid cell types (57–59). Conversely, defects in the Th17 cell differentiation axis may predispose the host to bacterial and fungal infections at mucosal surfaces (60). Th17 cells mediate their pro-inflammatory function by (i) recruiting neutrophils, (ii) activating macrophages, and (iii) enhancing Th1 effector cells (54). Much of the inflammatory damage previously ascribed to type 1 response is now thought to depend on IL-17 and IL-23 (the cytokine responsible for supporting Th17 response *in vivo*) (58).

CD4+ Th17 cells have been recently identified in borderline cases of leprosy (61), which highlighted their importance in infectious diseases as well. A persistent and very relevant concept is that an imbalance between Th17 and Treg cell function may be critical in the immunopathogenesis of many disease states (62). This concept is highlighted in a leprosy-specific study, where IL-10+ produced by Treg cells in BL/LL patients correlates significantly with polarized immunity highlighted by lesser IL-17 by CD4+ T cells in the same group. Blocking of IL-10/TGF-β resulted in the reversal of effector immune response (IL-17) in BL/LL with higher frequency of Th17 cells (63). This indicates that by negating the influence of suppressive cytokines we can successfully gain back immune responsiveness. The presence of Th17 cytokines (IL-6, IL-17, and IL-23) *in vitro* results in reduction of FoxP3 expression on Tregs simultaneously, possibly leading to increase in IL-17-producing CD4+ cells in BL/LL (63). This further suggests that the generation of antigen-specific Treg cells is very much dependent on the environment of cytokines they are exposed to. Hence, these cells may be targeted for reversal of effector response in BL/LL patients proving to be an important mode of immune modulation in the immunocompromised hosts to revive the immune response.

An imbalance in Treg and Th17 populations has also been observed in patients with leprosy reactions (53, 64). Studies done in biopsies from T2R patients showed a decrease in Tregs and associated cytokines, TGF-β and increase in cells producing IL-6, IL-21, and IL-17. On the other hand, T1R patients are showing the opposite trend with increased Tregs and reduced IL-17+ cells. This increase in inflammatory cytokines along with downregulation of Tregs may be responsible for the lesional inflammation characterizing T2R reactions.

## The Programmed Death-1(PD)-1-Programmed Death Ligand-1 (PD-L1) Pathway

T cell responses during parasitic infections are tightly controlled by co-stimulatory or co-inhibitory molecules. It is well known that interactions between PD-1 and its ligand, PD-L1 can inhibit the effector functions, such as proliferation, cytokine production, and survival of the T cells, thus balancing the tolerance, autoimmunity, infection, and immunopathology (65, 66). On infection with *M. tuberculosis*, protective T cells are generated in the infected host. However, T-cell-mediated immunity does not easily eradicate these bacteria because they have evolved effective strategies to overcome the host defense mechanisms (67). Studies have identified various virulence-associated genes and intracellular survival mechanisms of mycobacteria (68). The PD-1 signaling pathway is activated during persistent infection with various microorganisms and contributes to the impairment of protective immunity (69–71). A recent study showed that *in vitro* blockade of PD-1 signaling with the specific antibody enhanced IFN-γ production by T cells of TB patients on stimulation with *M. tuberculosis* antigen (72). In pulmonary TB patients, inhibiting this signaling pathway rescues *M. tuberculosis*-specific IFN-γ producing T cells from apoptosis (73). Similarly, persisting infection with pathogens like *Helicobacter pylori* and *Porphyromonas gingivalis*, showed elevated expression of PD-L1 on gastric epithelial cells and monocytes, suggesting a potential involvement of PD-L1 in promoting chronic infections (74).

Leprosy-specific studies show reduced expression of the positive signaling co-stimulatory molecules, CD28 and CD86 on T-cells, consistent with the LL anergy, in contrast to TT patients which displayed increased expression of the negative signaling molecules CD152 and PD-1 (75). This may represent a probable means of modulating an exacerbated immune response and avoiding immunopathology. However, another recent study in leprosy reveals elevated surface expression of PD-1 on T cells, NKT, and Treg cells and its ligand PD-L1 on APCs, such as monocytes and B cells, in BL/LL as compared to BT/TT leprosy patients (63). The authors have demonstrated that the PD-1/PD-L1 pathway preferentially suppress IFN-γ against TNF-α in BL/LL which is touted as the designate cytokine for generating protective immune response in the immunosuppressed host. This may also be one of the contact-dependent mechanisms utilized by Treg cells for immune suppression of effector T cells. These findings raise the possibility that the antigen-specific T-cell response is impaired by several inhibitory mechanism(s), thereby allowing mycobacterial persistence.

## Conclusion

**Figure 2:**
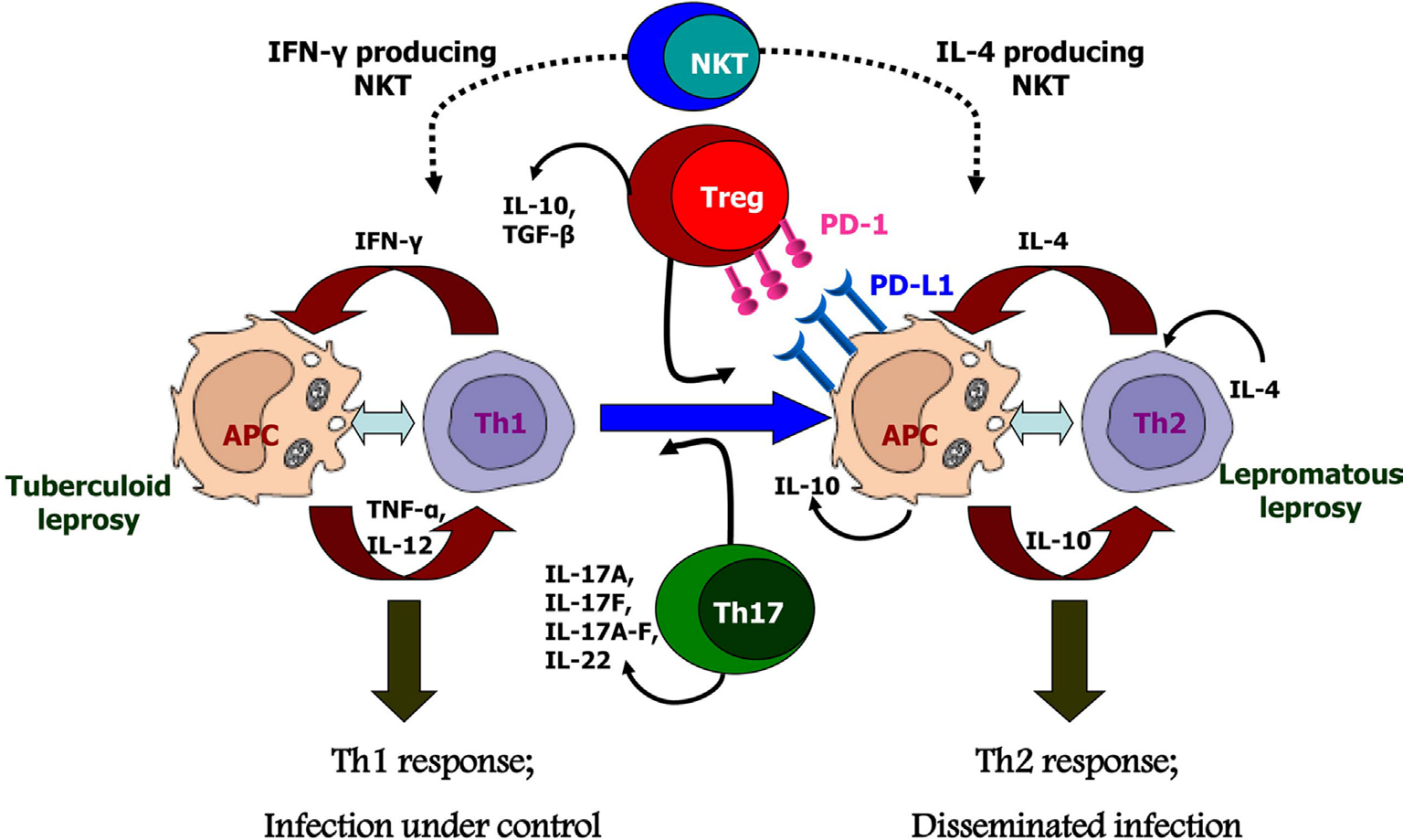
Possible causes for polarized host immunity in tuberculoid type (TT) vs. lepromatous leprosy questioning the well-established Th1–Th2 paradigm. Natural killer T cells which are initial responders producing either Th1 cytokines like IFN-γ or Th2 cytokines like interluekin-4 depending on the basal cytokine response of the host. Tregs cells which are predominantly suppressive in nature and produce cytokines IL-10 and TGF-β, along with increased expression of programmed death-1 and its ligand, programmed death ligand-1 on antigen-presenting cells; these cells are found in significant numbers in lepromatous leprosy patients. Th17 or T helper 17 cells which produce the cytokines IL-17 and IL-22 demonstrate inflammatory phenotype and are, therefore, found in increased numbers in TT leprosy patients.

However, it needs to be emphasized that no single mechanism of suppression can account for the kind of *M. leprae*-specific T cell anergy evidenced in LL. The diversity of effector mechanisms characteristic of NKT affords versatility capable of restraining diverse types of inflammatory responses in different tissues. Likewise, both Tregs and Th17 cells can exert beneficial as well as pathogenic effects depending on the physiology of the infected host. Other cell subsets, such as Th9 (76) or γδ T cells, have also been identified in leprosy patients, but their exact roles have not been defined till date. The intricate mechanisms governing differentiation and functions of these pro- and anti-inflammatory cells are yet to be discerned and pose major challenges ahead. Therefore, in conclusion, we can state that as seen in other chronic granulomatous diseases, NKT and Treg cells along with Th17 and the PD-1-PD-L1 pathway play crucial roles in the outcome of the host–parasite interactions in leprosy (Figure 2). Providing a balanced level of function for these cell subsets is the key to achieving an appropriate level of parasite control without inducing immunopathology. This would be a major goal in the management of this still-challenging infectious disease.

## Author Contributions

Author has planned and structured the review article.

## Conflict of Interest Statement

The authors declare that the research was conducted in the absence of any commercial or financial relationships that could be construed as a potential conflict of interest.
